# Vitamin E, Turmeric and Saffron in Treatment of Alzheimer’s Disease

**DOI:** 10.3390/antiox5040040

**Published:** 2016-10-25

**Authors:** Nur Adalier, Heath Parker

**Affiliations:** 1 Koc University Medical School, Istanbul 34450, Turkey; nadalier14@ku.edu.tr; 2 Chair of Internal Medicine & Pediatrics, Alabama College of Osteopathic Medicine, Dothan, AL 36303, USA

**Keywords:** Alzheimer’s disease, vitamin E, turmeric, saffron

## Abstract

Alzheimer’s disease (AD) is a growing epidemic and currently there is no cure for the disease. The disease has a detrimental effect on families and will strain the economy and health care systems of countries worldwide. The paper provides a literature review on a few ongoing possible antioxidant therapy treatments for the disease. The paper highlights use of vitamin E, turmeric and saffron for an alternative antioxidant therapy approach. Clinical studies report their therapeutic abilities as protective agents for nerve cells against free radical damage, moderating acetylcholinesterase (AChE) activity and reducing neurodegeneration, which are found as key factors in Alzheimer’s. The paper suggests that future research, with more clinical trials focused on more natural approaches and their benefits for AD treatment could be worthwhile.

## 1. Introduction

Alzheimer’s disease (AD) is a neurodegenerative disease, characterized by the variable deterioration of the cognitive abilities of those affected. Those who have Alzheimer’s disease begin to lose their cognitive abilities, including memory, speech, emotions, and personality. Alzheimer’s disease is a growing epidemic. In 2016, 44 million people suffer from Alzheimer’s and related dementia worldwide [1]. It is estimated that by 2050, 1 in 85 people worldwide will be afflicted with Alzheimer’s disease [2]. Alzheimer’s disease is currently the sixth leading cause of death in the United States [3]. In 2013, the journal Neurology published a study that estimated that by 2050, the number of people diagnosed with Alzheimer’s disease will be approximately 14 million in the US [3]. The AD epidemic strains the economy and health care systems of countries worldwide. Moreover, the disease has a detrimental effect on families physically, mentally, and financially. Scientists estimate the cost to be $226 billion in 2015 and up to $1.1 trillion by 2050 [4]. Ultimately, a cure for Alzheimer’s disease would be ideal; but if not, it is crucial that an efficient and cost-effective treatment plan be developed.

AD is a progressive neurodegenerative disease characterized by brain cell death that happens over a variable course of time. Although the pathogenesis of the disease is still not fully understood there is a growing consensus that behind the complex molecular mechanism appears to be the accumulation and aggregation of protein fragments. Amyloid-Beta (Aβ), known as amyloid plaques on the blood vessels and the accumulation of intracellular neurofibrillary tangles (tau) that block neurotransmitters and alter metabolism of iron, cause the destruction of nerve cells that accompanies Alzheimer’s [5,6,7,8,9,10,11,12]. It is reported that increased Aβ levels in AD patients reduce plasmalogen levels [13], increase the formation of reactive oxidative species, and slowly obstruct cerebral function [14,15]. Plasmalogens protect cells against the damaging effects of reactive oxygen species. Plasmalogens are susceptible to oxidative stress and function as antioxidants [16]. It has also been detected that plasmalogens, key structural phospholipids in neuronal membranes, are decreased in the brain of AD patients [16,17].

Clinical studies also indicate that oxidative stress is a crucial factor in AD [18,19]. Oxidative stress is a pathological state that indicates an imbalance between the production of reactive oxygen species (ROS) and the antioxidant defenses. Oxidative stress precedes Abeta production, promotes the production and aggregation of Abeta, and enhances the polymerization of intracellular neurofibrillary tangles [20]. Oxidative stress increases during ageing and is considered as a critical pathogenic factor for the onset and progression of AD [21,22,23,24,25]. Oxidative stress triggers oxidation of biomolecules leading to cellular damage [26] and has been suggested as one of the biochemical markers for the diagnosis of AD. Products of oxidative stress may diffuse into the blood and evaluating them can contribute to diagnosis of AD [27]. Although the sources of free radicals that cause oxidative stress are not fully known, some clinical studies suggest that mitochondria play an important role in the generation of free radicals, leading to oxidative damage to lipids, proteins, and nucleic acids [28]. Studies also suggest that an increase in polyunsaturated fatty acids (PUFAs) and high levels in redox metal ions could cause oxidative stress [19]. Elevated concentration of lipid peroxidation markers are detected in the brain tissue, cerebrospinal fluid and plasma of AD patients. Enhanced lipid peroxidation is linked to oxidative stress and Abeta formation [19,29,30].

A clinical study to determine the relationship between oxidative stress and cognitive performance found that high lipid peroxidation and decreased antioxidant defenses may be present early in cognitive disorders [31]. Due to the importance of the oxidative stress in AD, studies on the administration of antioxidants in AD treatment have been in progress. Antioxidants help to enhance cholinergic neuronal function or promote neuroprotective effects [32]; to convert free radicals into less reactive compounds, and provide protection for cellular components critical in the functioning of the body system [33]. PubMed, Scopus and Web of Science review on the oxidative stress studies indicate that markers of lipid peroxidation increased in AD while copper metabolism is dysregulated and total antioxidant capacity is decreased, resulting in oxidative stress that triggers neurodegeneration [34]. Applying antioxidant therapy (especially curcumin and lipid soluble vitamin E) is suggested as one of the various neuroprotective strategies in responding to oxidative stress especially in primary prevention stage [34,35].

Currently Alzheimer’s is considered an incurable disease [36]. Recovering the terminally damaged neuronal networks may not be possible because it is challenging to rejuvenate or replace dead nerve cells. Though a cure for the disease has not been found there are treatments that may help with both cognitive and behavioral symptoms of the disease [37] as well as prevention. Neuroprotective antioxidant treatment is one of the suggested treatment strategies in AD.

## 2. Vitamin E

Vitamin E is a potent antioxidant that may have beneficial effects in Alzheimer’s in dealing with oxidative stress [38,39] and Aβ-associated free radicals [40]. Vitamin E is a group of eight different compounds that consist four forms of tocopherols and four forms of tocotrienols attached with alpha (α), beta (β), gamma (γ), and delta (δ) vitamers which collectively support antioxidation in the body. Clinical studies reported various findings on the benefits of different forms of vitamin E. When considering eight different forms of vitamin E, some clinical studies suggest that α-tocopherols and γ-tocopherols are the two forms that are most associated with a slower rate of cognitive decline [41]. It was reported that α-tocopherol is the most bioavailable antioxidant form of vitamin E and is helpful in AD [21,42,43,44]. Alpha-tocopherol was found as protective against lipid peroxidation [44] and decreased in plasma in patients with mild AD [43].

A clinical research study investigated the influence of α-, γ- and δ-tocopherol compounds on Aβ production and degradation in neuronal cell lines [45]. Researchers found that all forms of tocopherol enhanced the Aβ production and decreased the Aβ degradation. Tocopherols increase their protein levels. Researchers reported significant differences between the tocopherol compounds. They found while α-tocopherol had minor effects on Aβ production δ-tocopherol was the most effective in increasing Aβ generation [45]. In summary, researchers concluded that vitamin E has antioxidative effects and tocopherol helps increase the amyloid-β level [45]. Researchers suggested further studies on the potential role of various vitamin E forms in treating AD, and identification of the specific vitamin form without amyloidogenic potential. Although there are relatively fewer studies on tocotrienols, it has been found that tocotrienol molecules offer more powerful antioxidant capacity and blood cholesterol-lowering properties compared to tocopherol molecules due to the differences of their molecular structure [46]. Tocopherol molecules have a long tail with no double bonds whereas tocotrienol molecules have a short tail with three double bonds which enables tocotrienol move efficiently around cells, better cleanse the arteries of accumulated cholesterol and remove the plaque in arteries [46]. Some studies found that γ-tocotrienol has a higher antioxidant effect than α-tocotrienol [47,48]. A clinical study investigated the relations of α- and γ-tocopherol and the amyloid load and neurofibrillary tangle severity in AD patients [47]. Researchers found that γ-tocopherol concentrations were related with lower amyloid load (β = −2.10; *p* = 0.002) and lower neurofibrillary tangle severity (β = −1.16; *p* = 0.02) whereas α-tocopherol was associated with higher amyloid load. Researchers concluded that γ-tocopherol could be important for the neuroprotection of the brain [47].

A number of clinical studies cited the benefits of vitamin E therapy in delaying the progression of AD [49,50,51,52,53,54,55]. To determine the effect of vitamin E (alpha tocopherol) on AD, a clinical trial on patients with mild to moderate AD was conducted. Patients who were given 2000 IU/day of alpha tocopherol compared with a placebo showed a slower functional decline. The researchers reported that vitamin E (alpha tocopherol) could be beneficial in slowing functional decline in mild to moderate AD [54]. A high intake of vitamin E is linked to preventing or slowing the progression of the disease as vitamin E has been found to be a powerful antioxidant and have neuroprotective effects [49,51,52,53,55]. Antioxidants reduce neuronal death.

Scientists recognize oxidative stress to be a hallmark of AD, as it has been shown to play a role in altering cell signaling. Oxidative stress disrupts the signaling pathway, which causes tau hyperphosphorylation [52,56]. Thus agents possessing the ability to prevent oxidative damage may be a promising approach to treating AD [52]. Also, most prior research confirms that vitamin E treatment could prevent or reduce the oxidative stress-dependent brain damage [53]. Alphatocopherol, a major form of vitamin E, may possess the ability to reduce free radical mediated damage in the brain [54]. For instance, in a clinical study conducted on patients with mild to moderate AD, participants were administered either 20 mg/day of alphatocopherol (a form of vitamin E), Memantine, both, or a placebo [54]. The researchers found that participants given solely alphatocopherol had a slower deterioration than those given the placebo. There were however, no noteworthy differences between the memantine and the combination (memantine and alphatocopherol) groups. Participants using memantine or the combination group had a greater frequency of severe adverse events constituting mainly of serious infections [54]. Researchers concluded that alphatocopherol may have benefits for patients with mild to moderate AD in slowing the progression of the disease.

Lipid oxidation and oxidized DHA are suggested to be harmful to human health. Oxidized lipid intake or lipid peroxidation can contribute to the development of tumors and atherosclerosis. Clinical studies suggest lipid peroxidation can be partially counteracted by vitamin E intake [57,58,59,60].

Numerous studies show the efficacy of antioxidants such as vitamin E as a therapeutic agent [52,61,62,63]. Vitamin E is also found effective in managing acetylcholinesterase activity [53,64,65,66]. Ahmed (2012), tested the treatment of vitamin E, acetyl-l-carnitine, and alpha-lipoic acid on AD induced rats [53]. Brains with AD are characterized by increased acetylcholinesterase levels and reduced folic acid and vitamin B_12_ levels. The treatment was found to restore those levels to normal, like donepezil. They found the treatments might possess potential restoring effects. There is significant evidence that dietary supplementation may delay the progression of AD and other dementias, as the vitamin E treatment significantly restored acetylcholinesterase activity and increased the Na^+^/K^+^ ATPase activity. Though donepezil was shown to have the most prominent effect, vitamin E was not far behind. Both treatments increased acetylcholinesterase inhibitor and vitamin B_12_ levels. Some studies found that low levels of vitamin B_12_ might be linked to AD [66,67,68]. Vitamin E gave the most significant rise in insulin levels by 6.4% as compared to donepezil [52]. It is found that insulin may contribute to AD [69,70] as insulin may regulate the amyloid precursor protein and the production of amyloid-beta, one of the key factors that trigger AD. A clinical trial reported that increased levels of insulin influence learning and memory processes [70] and insulin resistance may increase AD. Some clinical studies suggest that high-dose supplementation of B_12_ might contribute to AD by lowering the homocysteine concentration, which was found as a risk factor in AD [71,72,73].

A population-based study was conducted on the relation between the consumption of antioxidants and long-term risk of dementia. The researchers used a sample of 5000 participants, 50 years and older, without dementia. The participants were given a dietary and lifestyle guideline and they were followed up for 9.6 years. Scientists found those who had a higher intake of vitamin E were associated with lower long-term risk of dementia. Thus vitamin E rich foods may possibly have the ability to reduce long-term risk of AD. It was found that those in the highest tertile, who had an average intake of 18.5 mg/day of vitamin E, were 25% less likely to develop dementia than those in the lowest tertile who took 9 mg/day [50].

High vitamin E levels in plasma have also linked to reduced risk of Alzheimer’s in advanced age [74]. Research on the effect of vitamin E on cholesterol metabolism also has promising results [75]. Although the impact of cholesterol in neurodegeneration was not conclusive in some studies [76,77] a number of studies found cholesterol as a risk factor for Alzheimer’s disease [78,79,80]. It has been reported that cholesterol causes higher levels of amyloid plaque deposits in the brain which contributes to Alzheimer’s [79].

It has been found that vitamin E reduces cholesterol-related oxidation in the heart, liver and kidneys [81]. To determine the relationship between high cholesterol-induced oxidation and vitamin E supplements in the heart, liver and kidney, researchers gave high cholesterol with and without vitamin E supplement and regular diet, over two-month and four-month periods to four different groups of rabbits. The hearts, livers and kidneys of the sample were tested for malondialdehyde (MDA)—a biomarker for oxidative stress—and chemiluminescence (CL)—a measure of oxidative stress and antioxidant reserve.

Researchers found that the increase in the heart MDA levels in the vitamin E group was 2.42-fold compared to the 3.16-fold increase in the two-month cholesterol diet and 14.65-fold increase in the four-month cholesterol diet. Researchers found that vitamin E helped to reduce the MDA in the heart by more than 83 percent; in the kidney up to 71 percent. The liver MDA levels were similar to those of the heart. As for CL, researchers found that vitamin E helped to keep antioxidant reserve at control levels in the heart and increased the antioxidant reserve in the liver. Researchers concluded that vitamin E slows the progression of oxidative stress [81].

Research studied the type of oxidation and the duration and timing of use in affecting vitamin E’s ability to offset LDL (low density lipoprotein) oxidation [75]. It was found that vitamin E could provide an effective treatment for atherosclerosis caused by LDL oxidation if the oxidation is caused by free radical damage and if the vitamin E supplement is taken early on [75].

Although high plasma vitamin E is linked to better cognitive performance [32] there are still some studies that report some disadvantages [82,83,84,85,86,87,88,89]. For instance, the factors that may be involved with the intestinal absorption of vitamin E are not accurately available [82]. Its bioavailability, for instance, may be affected by various factors such as proteins and fat soluble micronutrients in the diet that may impact the absorption of vitamin E [82]. Still there is an ongoing research about the metabolization of vitamin E in the intestinal lumen and factors that may play a role in the absorption of vitamin E [82].

Also a number of studies found the potential pro-oxidative effects of vitamin E [83,84]. For instance, the pro-oxidative effects of high dose of vitamin E are linked with increased mortality [85] and incidents of heart failure [84,85]; α- and γ-tocopherol have pro-oxidant activity toward HDL (high density lipoprotein) [86,87] and α-tocopherols on type 2 diabetes patients, with respect to acute hyperglycaemia [88]. Clinical studies that involve vitamin E supplements also found that tocopherol could facilitate the formation of free radicals and this pro-oxidant effect may impact the increase in fatal myocardial infarctions [85,89].

Some studies on the effect of vitamin E were inconclusive. In a study over a one-year period, vitamin C (1000 mg/day), and vitamin E (400 IU/day), were given to AD patients taking cholinesterase inhibitors. The supplementation decreased lipid peroxidation markers in CSF (cerebrospinal fluid); however, no significant difference was observed in cognition of the patients [51]. In a clinical study on 769 subjects, researchers found no significant differences in the progression of AD between the placebo group and the group supplemented vitamin E [90]. The study did not report any specific form of vitamin E or the composition of vitamin E supplement.

The literature reveals a lack of consensus on the level of efficacy of vitamin E treatment. Some studies claim that it only benefits patients with mild to moderate Alzheimer’s disease [44,54]. Several studies reveal that the dosage level has no effect in delaying the neurodegeneration in patients with more advanced AD [49,91]. Also, scientists dispute whether the effect of vitamin E may differ based on the patient’s response to its antioxidant properties [51]. Therefore vitamin E acts as an antioxidant and prevents or slows decline in only some patients [51].

Some vitamin E studies reported no clinical benefit. Over the three years of the study, the scientists studied to assess the benefit of vitamin E in AD patients. For the first six weeks 1000 IU/daily initial dose of vitamin E was given and the dose was increased to 2000 IU/daily after six weeks. The study demonstrated that no significant effect of vitamin E on AD patients [91].

Various meta-analyses informed on the controversial effects of the relationship between vitamin E and mortality. A meta-analysis of 19 papers that included 135,967 patients aged 47–84 years informed that vitamin E supplementation at doses above 400 IU/day is associated with a small increase in mortality from all causes [85]. Another meta-analysis between 1988 and 2009 that included 246,371 subjects, informed that supplementation with vitamin E appears to have no effect on mortality from all causes at doses up to 5500 IU/day [92]. In a 1997 clinical study over two years, 341 AD patients were given a placebo, vitamin E (2000 IU/day dl-alpha-tocopherol), a monoamine oxidase inhibitor (selegiline), or vitamin E and selegiline in random groups. Although deterioration slowed down in those taking vitamin E separately or together, mortality increased in those who take vitamin E [93]. Another clinical study aimed to assess the association of vitamin E (at a dose of 2000 IU) alone, or in combination with a cholinesterase inhibitor (ChEI), with mortality from all causes on 847 AD patients [49]. The research showed that patients who have daily intake of vitamin E tended to survive longer than those taking acetylcholinesterase inhibitors. The scientists administered 2000 IU/day of vitamin E, a combination of vitamin E with cholinesterase inhibitors, or a placebo to the patients [49]. Researchers found the hazard ratio of vitamin E to be around 0.71 and 1.2 for the cholinesterase inhibitor. The findings did not indicate that a high dose vitamin E supplementation was linked to increased mortality.

Some scientists have questioned the safety of the vitamin E dose level as some studies may have found it linked to increased mortality [94,95]. Vitamin E may have adverse reactions such as nausea, diarrhea, fatigue, headache or bleeding. Normally an adult may consume up to 1500 IU/day of vitamin E. Though the dosage level has been shown to significantly delay the progression in patients with mild to moderate AD, the given dosage of vitamin E had no significant effect on delaying the neurodegeneration of patients with more severe AD [49,55,74]. The vitamin E treatment may not have been effective for more severe AD because of the dosage level, as it was not drastically higher than the average adult intake. Nevertheless, antioxidant vitamins are currently commonly used for patients with AD. Researchers found the intake of a vitamin E and C supplementation for more than a month caused an increase in the cerebrospinal fluid and a decrease in the cerebrospinal fluid lipid peroxidation levels. The scientists performed a clinical trial studying the effect of vitamin E and C supplementation for a year. The patients were either taking a cholinesterase inhibitor with 1000 mg/day vitamin C and 400 IU/day vitamin E or solely cholinergic medication [51]. The results indicate no significant difference between the group taking the supplementation and the control group. This finding may be attributed to the low dosage level administered. There was however a small antioxidant effect observed in the cerebrospinal fluid [51].

## 3. Turmeric and Saffron

Turmeric (curcumin) and saffron, natural spices commonly used in South Asian cuisine such as curry, can be considered as promising alternative treatments [35,96,97].

These spices have minor adverse effects and several studies show them to be equally as effective as Donepezil [98,99,100,101]. A review of the benefits and adverse effects of the two treatments reveals the alternative treatment, saffron and turmeric, to be the more promising solution to treating those with Alzheimer’s disease [102].

Alzheimer’s disease is an epidemic in industrialized western nations. The incidence rates for Alzheimer’s disease is much higher in the US and European nations than in East Asia [103,104]. Ballabgarh, a rural town in Northern India, is reported to have the lowest rate of Alzheimer’s disease in the world, with only 0.62% of people above the age of 55 and 1.07% above the age of 65 with the disease [103]. Two studies that tested the Alzheimer’s disease incidence rate reported the incidence rate of people above the age of 65 in Ballabgarh with Alzheimer’s disease to be only 4.7 per 1000 people as compared to 17.5 in 1000 people in Monongahela Valley, Pennsylvania, US [103,104]. There is significant evidence that the spices consumed in the Indian diet may be the reason for the low incidence rates of Alzheimer’s disease.

### 3.1. Turmeric Studies

Turmeric’s healing ability can be attributed to the chemical composition of turmeric. Though the spice contains over 235 compounds, the major bioactive ingredients are curcuminoids, the most common being curcumin [100]. In traditional South Asian medicine, turmeric (curcumin) is used as medicine to relieve a plethora of ailments such as wounds, gallstones, cramps, and Alzheimer’s disease due to its anti-inflammatory and antioxidant properties [96,105]. A study on the relationship between curry consumption and cognitive function in the elderly found that elderly Asians who “often” (once a month or more) or “occasionally” (one or more in six months) consumed curry had superior cognitive function to those who “rarely” (less than one in six months) consumed curry [98]. Their considerably higher MMSE (Mini-Mental State Examination) scores proved their superior cognitive function [98]. Those who “often” or “occasionally” consumed curry had the odds ratio of 0.51 and 0.62 for Alzheimer’s disease whereas those who “rarely/never” had the odds ratio of 1 [98]. Scientists reported the incidence of Alzheimer’s disease for Indians aged 70–79 to be four times lower than the rate in the US (98). Thus they concluded that turmeric enhances neurocognitive functions of Alzheimer’s patients.

A growing body of clinical research suggested curcumin is effective in AD as an antioxidant, anti-inflammatory therapeutic agent that improves the cognitive functions [97,106,107,108]. Clinical studies suggest that curcumin could be effective in decreasing oxidative damage [109,110], reducing inflammation in brain microglial cells [97,111], reversing neurodegeneration resulting from Aβ production [112,113], preventing β-amyloid protein formation [114,115], decreasing beta-amyloid plaques [107,114,116], inhibiting Aβ aggregation [117,118] and blocking cholesterol formation [119]. A clinical study on AD mice showed that AD mice that were fed low doses of curcumin had a 40% reduction in the level of beta-amyloid compared to those not fed curcumin [114]. Another clinical vitro study has found curcumin to possess the ability to prevent neuronal damage in the brain by impeding the formation of the amyloid beta plaques, thus reducing the number of plaques in the brain [104]. In a four-week clinical trial with healthy subjects [120], 80 mg/day of lipidated curcumin was given to assess the health benefits. The study reported the level of Aβ(1–40) in plasma was significantly reduced.

Altered metabolism of iron is also considered a critical factor in AD pathology. The scientists suggested that cholesterol-enriched diet may increase body iron deposition that may lead AD. Administering various metal chelators that will help prevent free radical reactions could be helpful in treating the disease [8,121,122]. Curcumin was found to have a high binding affinity for iron and copper, that may work as an iron chelator in AD [123,124]. In a clinical study mice were fed curcumin-supplemented diet and researchers observed a decline in levels of ferritin protein in the liver. The study reported that curcumin has properties of an iron chelator as it modulates proteins of iron metabolism in cells [125]. In a two-month clinical study on rats the effect of curcumin on iron overload-induced lipid peroxidation and anti-oxidant depletion was analyzed [126]. The study reported a significant reduction in both iron accumulation and endogenous anti-oxidant activities in liver and spleen of the curcumin fed rats. The researchers concluded that curcumin was effective in restoring the decrease in the hepatic and splenic antioxidant activities [126].

In a study to measure the effect of curcumin (turmeric) on focal ischemia of rats, scientists found that an injection of 1–2 mg/kg, i.v. of curcumin bettered the neurological deficit and reduced the water content in the brain [99]. Also, another study done using transgenic mice with several beta-amyloid plaques found that after administering 5 mg/day of turmeric extract for six months, the accumulation of beta amyloid plaques and tau phosphorylation was significantly reduced [100].

### 3.2. Saffron Studies

Saffron (*Crocus sativus*), a spice that also possesses therapeutic abilities, has been identified as a memory-enhancing agent [101]. A number of studies have investigated saffron’s therapeutic effects. Virgo and clinical studies reported that saffron demonstrated effective antioxidant [127] and anti-inflammatory [128,129] and antiamyloidgenic abilities.

A 22-week double blind controlled trial was done to compare *Crocus sativus* (Saffron extract) with the cholinesterase inhibitor donepezil (Donepezil) as treatments for subjects with mild to moderate Alzheimer’s disease. Participants in the clinical trial were randomly administered either a 30 mg/day capsule of saffron or 10 mg/day of donepezil [101]. After the 22-week study, it was found that saffron had a similar effect in the improvement of cognitive function in subjects with Alzheimer’s disease as donepezil (Donepezil), due to the acetylcholinesterase frequency being at a similar level [101]. The side effects of both treatments did not differ drastically but overall more vomiting, dizziness, fatigue, and nausea occurred with subjects taking donepezil. Subjects taking saffron had slightly more cases of dry mouth and hypomania. The study concluded that *Crocus sativus* was effective in treating mild-to-moderate Alzheimer’s disease due to its “antioxidant and antiamyloidgenic” abilities, thus being able to inhibit the aggregation and deposition of the beta-amyloid plaques [101].

Saffron is also reported to be benefical as a protective agent against toxicities [130] and in moderating acetylcholinesterase (AChE). AChE is associated with β-amyloid plaques and neurofibrillary tangles, a hallmark of AD [131]. In an in vitro enzymatic and molecular docking study on natural remedies that inhibit AChE, saffron extract was found to moderate AChE inhibitory activity by 30% [131]. In another study saffron extract treated rats over a period of 21 days reported that they had significantly higher levels of lipid peroxidation products and antioxidant enzymes and decreased plasma levels of corticosterone. The study concluded that saffron could be effective in impairment of learning and memory and improving the oxidative stress damage to the hippocampus caused by chronic stress [132].

In another study on the effect of saffron, on learning and memory loss and the induction of oxidative stress animals were treated with two doses of saffron extract (5 and 10 μg/rat) for a week [132]. After the seven days, treatment oxidative stress markers were measured. It was found that in the saffron treated group spatial learning and memory impairment (*p* < 0.05) increased and the overall antioxidant reactivity capacity and antioxidant enzymes activity were improved (*p* < 0.01). Researchers concluded that that saffron extract can improve the degeneration in learning and memory and the oxidative stress parameters [132].

Using turmeric and saffron as a treatment for Alzheimer’s disease may be controversial, as they may not be considered as a conventional pharmaceutical drug. However, they have been shown to be an effective treatment. Numerous studies have found saffron and turmeric to possess the ability to treat patients with Alzheimer’s disease as effectively as the conventional treatment, Donepezil. These spices used, as treatments are a safer alternative because they are natural and have less adverse effects. Yet it is suggested longitudinal studies and more clinical trials to better underpin the therapeutic effect of curcumin on AD patients [102].

## 4. Conclusions

A growing body of clinical study shows vitamin E, saffron, and turmeric treatment as potent antioxidants in reducing neurodegeneration and treating AD patients. Clinical studies report their therapeutic abilities as protective agent in protecting nerve cells from free radical damage and in moderating acetylcholinesterase (AChE) activity, associated with β-amyloid plaques and neurofibrillary tangles, which are found as key factors in Alzheimer’s. However, there is still more research to be done. For instance, additional research testing higher dosage ranges for vitamin E treatments needs to be conducted. Research testing the long-term efficacy of vitamin E treatments in AD patients with different levels of severity needs to be done since previous studies that administered low dosages of vitamin E did not show any effect on the neurodegeneration of AD.

Therefore, in future research turmeric and saffron therapy and various antioxidant therapies should be tested using different methods such as in vivo experiments and clinical trials while testing various dosage levels of the antioxidants. There should be more testing of both synthetic drugs and the natural substances for AD treatments. However, research should be directed towards more natural substances as they have a beneficial safety profile. It is noteworthy to be aware of side effect profiles of the natural substances and differences in manufacturing, as they may be synthetic, like the pharmaceutical drugs. In summary, the objective is to find a worldwide easily accessible and inexpensive treatment as the rate of AD is exponentially increasing.
